# Supplementary material for: Hydrophilic Interaction Liquid Chromatography Coupled to Mass Spectrometry and Multivariate Analysis of the De Novo Pyrimidine Pathway Metabolites
Source: Biomolecules. 2019 Jul 31;9(8):328. doi: 10.3390/biom9080328 (PMC6722987; doi:10.3390/biom9080328)

Supplementary Materials

Table S1. List of *dn*SP compounds.

Family	Metabolite	Polar Group			Rings
		–OH	O=	NH-	
Amino acid	Aspartic acid	2	2	1	
	Glutamine	1	2	2	
Nucleobase	Uracil		2	2	1
Nucleoside	Uridine	3	2	2	1
	Cytidine	3	1	3	1
Nucleotide mono-, di- y triphosphate	UMP	4	3	1	1
	UTP	6	5	1	1
	ATP	6	3		2
	CTP	6	4	2	1
	GTP	6	4	1	2
	UDP-glucose	8	4	1	1
Acids precursors/derivates of nucleosides	Carbamoyl aspartic acid	2	3	2	
	DHO	1	3	2	1
	Orotic acid	1	3	2	1

Table S2. Effects and significance of the variables evaluated on the response variables.

Column	Response	Temperature	Concentration	pH	Flow	Gradient
ZIC-p-HILIC	**R**	+	+	+	+	–
	**N**	+	+		–	+
	**h**	+	–	+	+	
	k	+	+	+	+	–
ZIC-HILIC	**R**	+				–
	**N**	–		+		
	**h**		–	+	+	
	k	–		+	+	–

The sign (+) indicates a positive effect on the response variable. The sign (–) indicates a negative effect on the response variable. The blank boxes indicate that the variable has no statistical significance on the response variables ($p > 0.05$).

Table S3. Optimal factor values of ZIC-p-HILIC and ZIC-HILIC columns.

COLUMN	FACTOR	RESPONSE			
		R	N	h	*k*
ZIC-p-HILIC	Temperature (°C)	40	40–41	38–42	≤35 y ≥45
	Additive conc. (mM)	≥15	≥15	≤6	10
	Flow (mL min^{-1})	≥0.35	≤0.25	≤0.25	≥0.35
ZIC-HILIC	Temperature (°C)	≤10	≤25	≥25	NS
	pH	5.2 y 6.8	≤5.4	≤5.2 y ≥5.8	NS

Table S4. Linearity, correlation coefficient (r^2), LOD and LOQ for ZIC-p-HILIC.

Compound	rt (min)	Diagnostic ion (*m*/*z*)	Linearity (ng mL^{-1})	r^2	LOD (ng mL^{-1})	LOQ (ng mL^{-1})
Aspartic acid	18.60	132.0302 $[M-H]^-$	1300–4100	0.994	820	1300
Glutamine	17.58	145.0619 $[M-H]^-$	1300–4100	0.994	800	1300
Uracil	3.55	111.0200 $[M-H]^-$	100–1600	0.992	50	100
Uridine	9.36	243.0623 $[M-H]^-$	100–1600	0.995	30	100
Cytidine	12.89	242.0782 $[M-H]^-$	400–2600	0.994	200	400
UMP	18.29	323.0286 $[M-H]^-$	600-2600	0.990	200	600
UDP-glucose	21.46	565.0477 $[M-H]^-$	600–2600	0.994	200	600
UTP	24.89	482.9613 $[M-H]^-$	2500–11000	0.991	2000	2500
ATP	23.50	505.9885 $[M-H]^-$	2500-7000	0.992	2000	2500
CTP	25.51	481.9772 $[M-H]^-$	2500–7000	0.990	2000	2500
GTP	ND	521.9834 $[M-H]^-$	ND	ND	ND	ND
Carbamoyl aspartic acid	21.11	175.0360 $[M-H]^-$	1800–3800	0.993	600	1800
Orotic acid	11.34	155.0098 $[M-H]^-$	300–1000	0.991	20	300
DHO	13.17	157.0255 $[M-H]^-$	50–1000	0.996	20	50

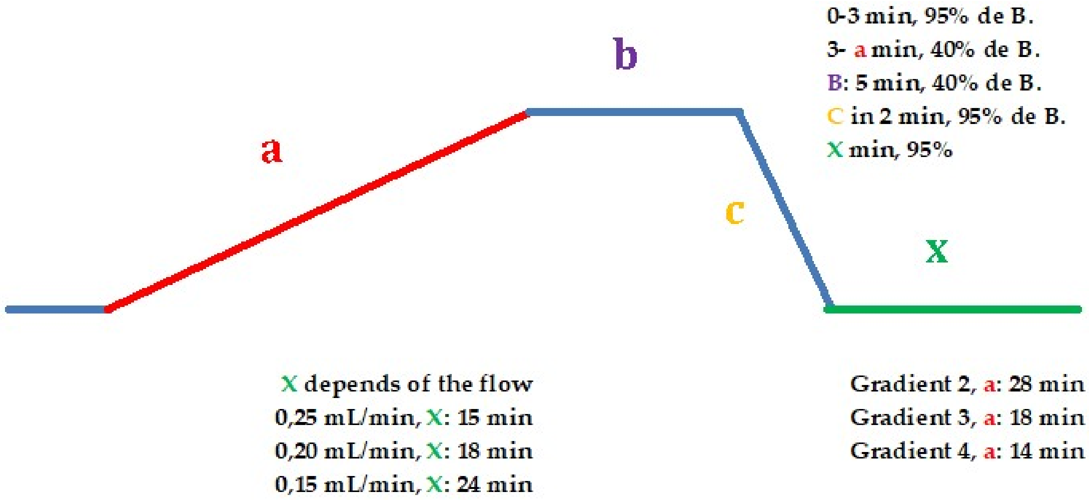

Figure 1. Chromatographic parameters.

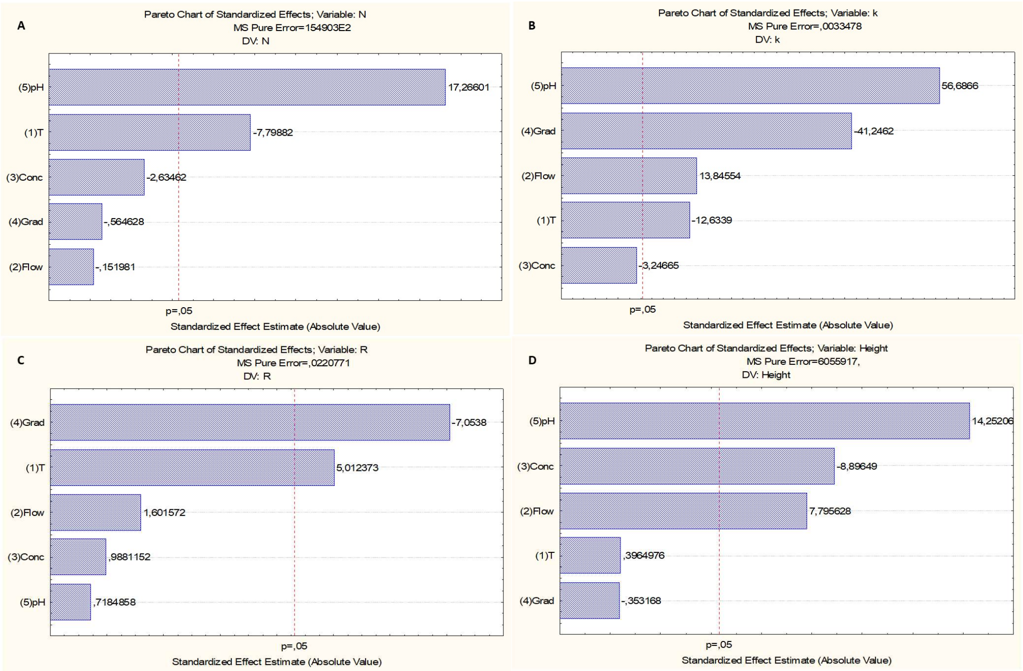

Figure 2. Pareto´s charts of standardized effects: (a) Efficiency (N); (b) Retention factor (*k*); (c) Resolution (R) and (d) Peak height (h).

Supplementary Figure 3. Extracted ionic current (EIC). ZIC-p-HILIC

Standard
rt (min)

Extracted ionic current

Uracil
3,55

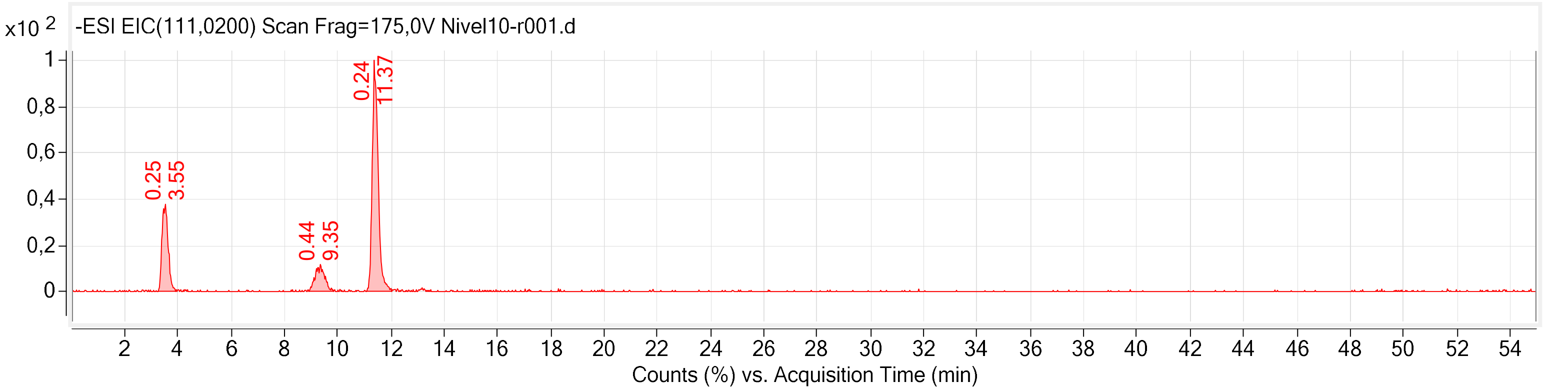

Uridine
9,36

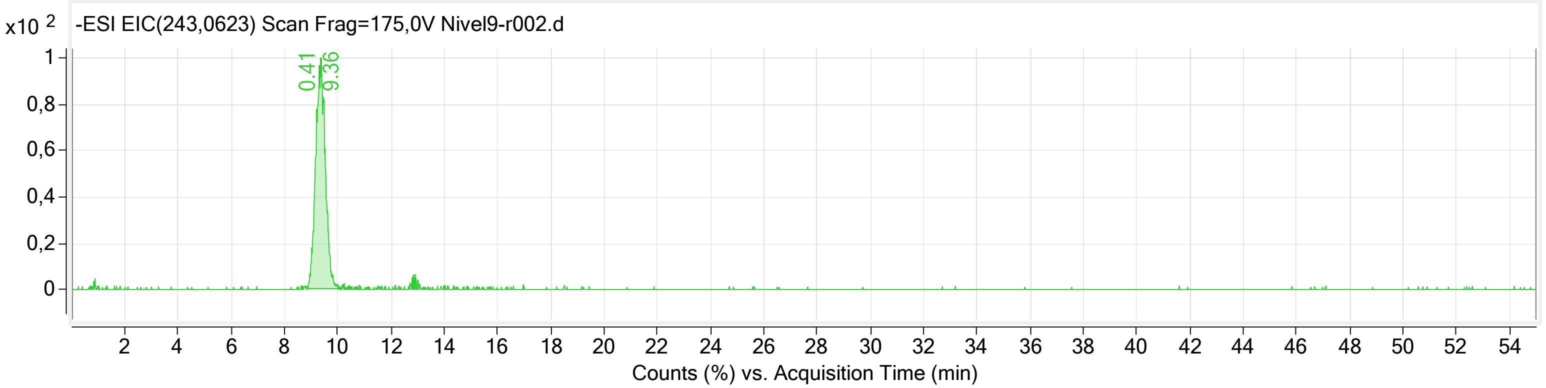

Orotic acid

11,34

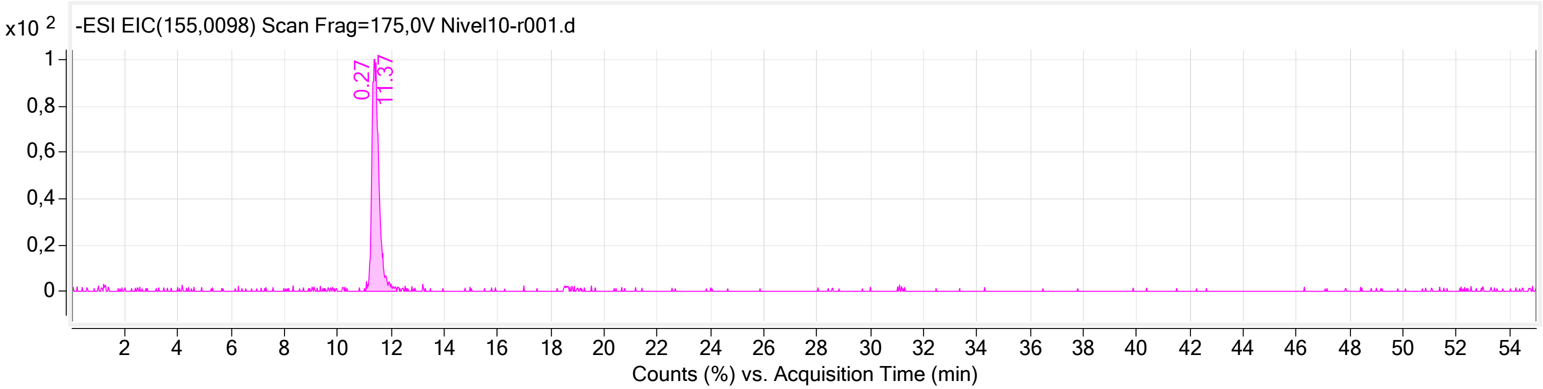

Cytidine

12,89

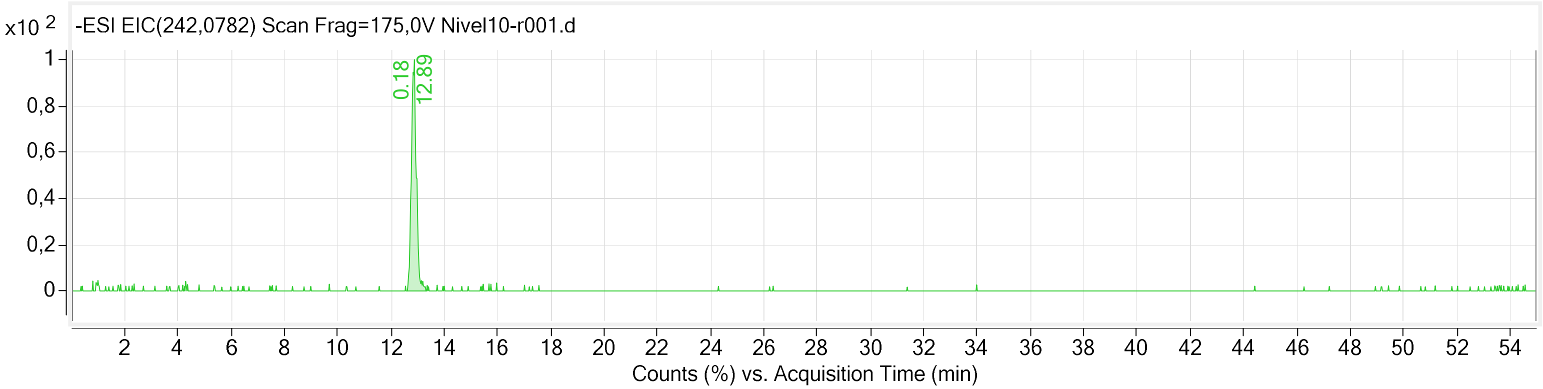

DHO

13,17

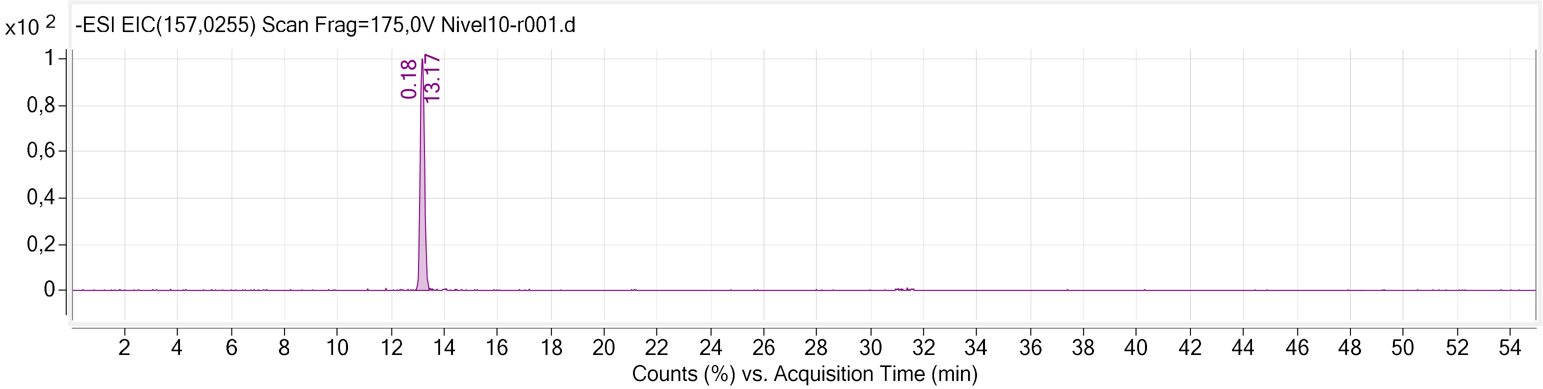

Glutamine

17,58

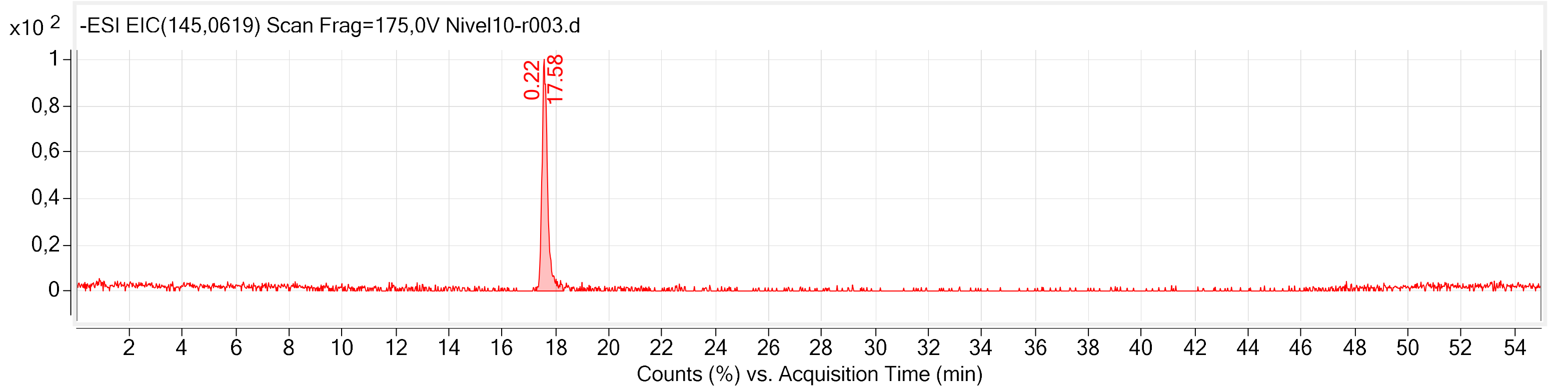

UMP

18,29

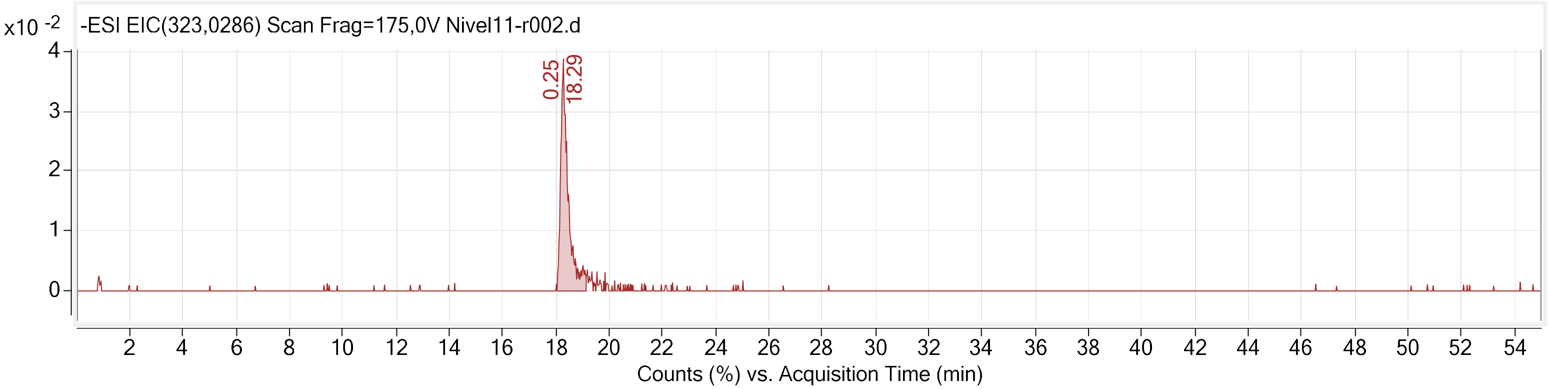

Aspartic acid

18,60

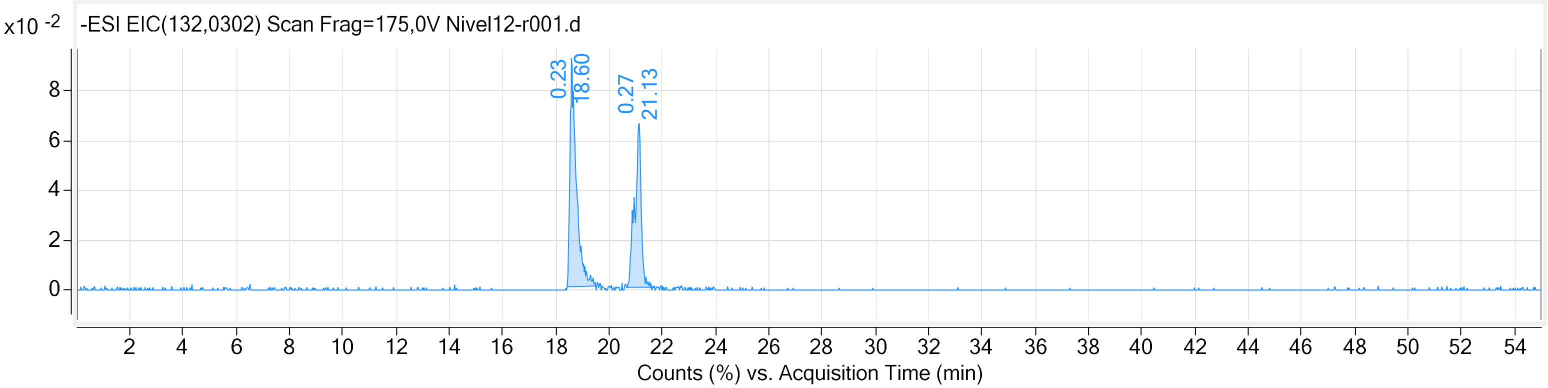

Carbamoyl aspartic acid

21,11

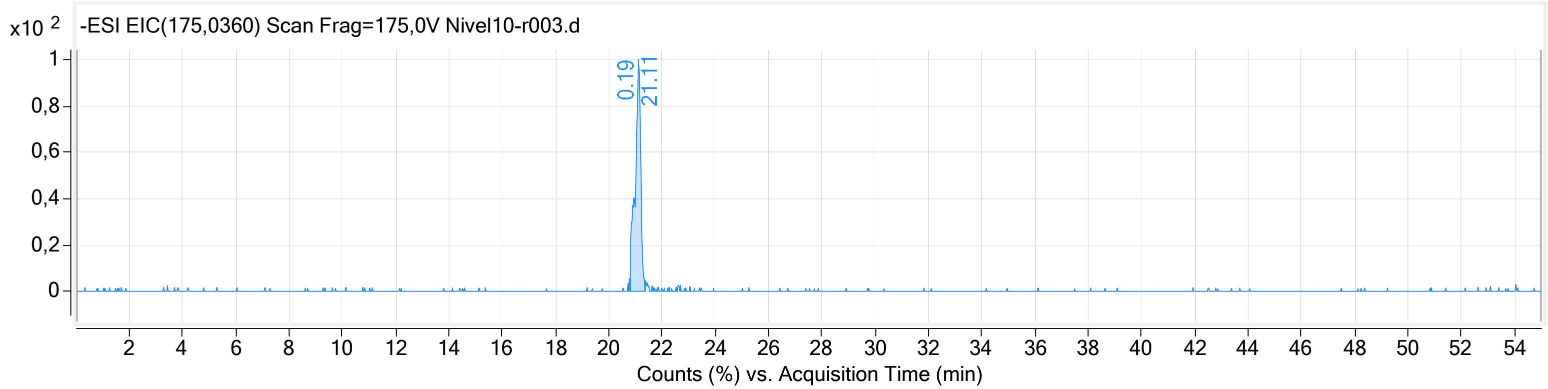

UDP-Glu

21,46

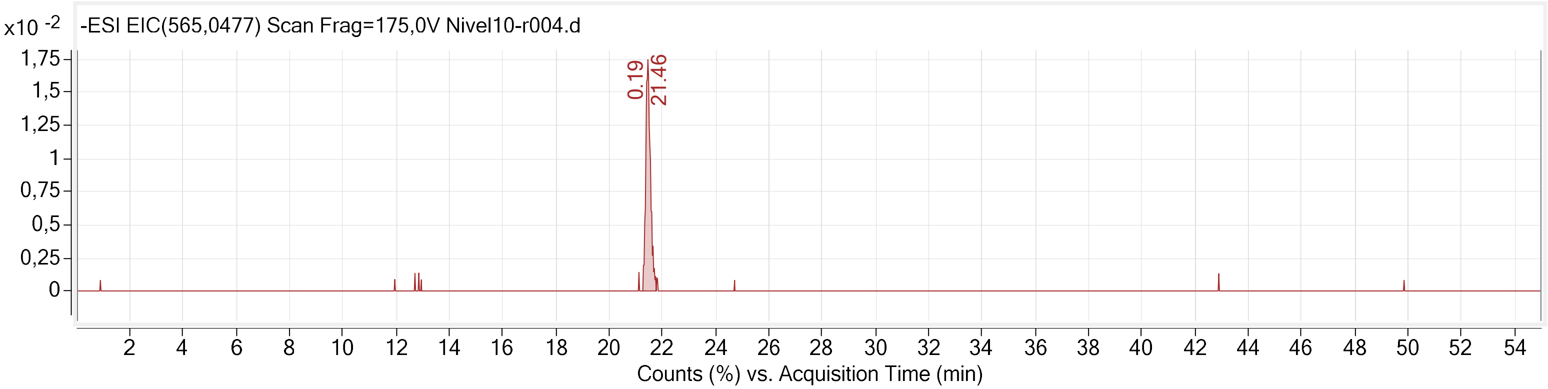

ATP

23,50

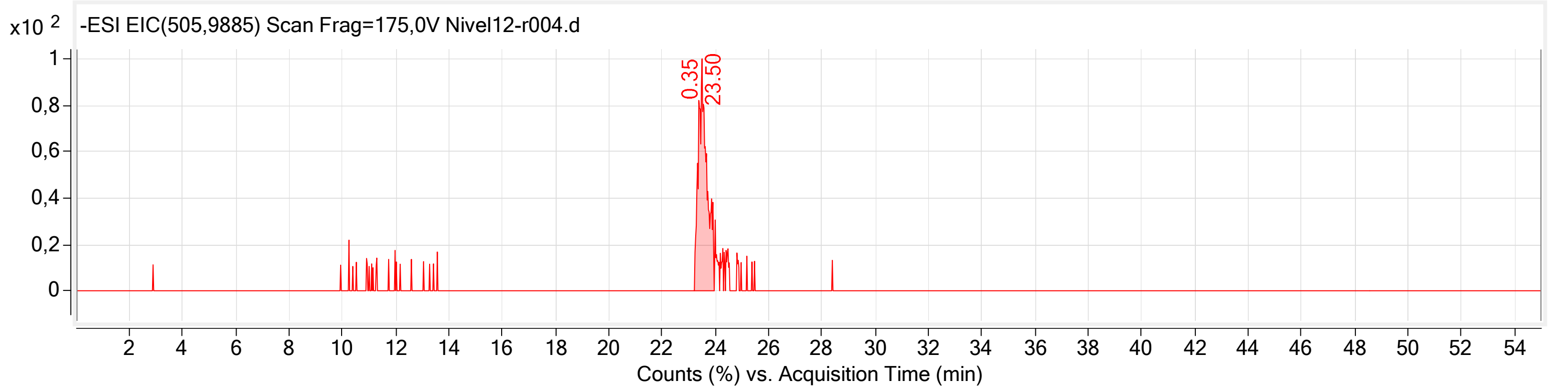

UTP

24,89

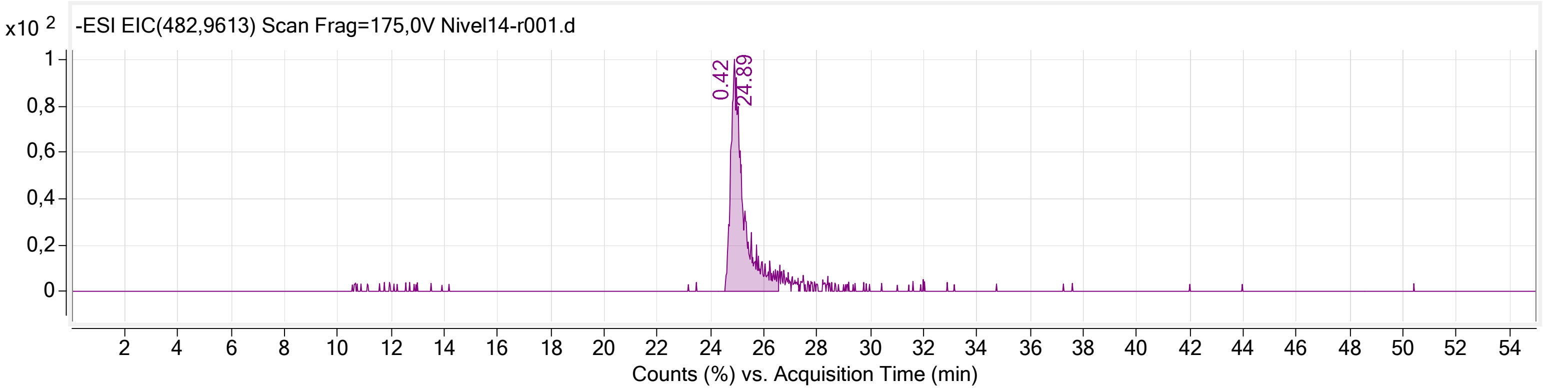

CTP

25,51

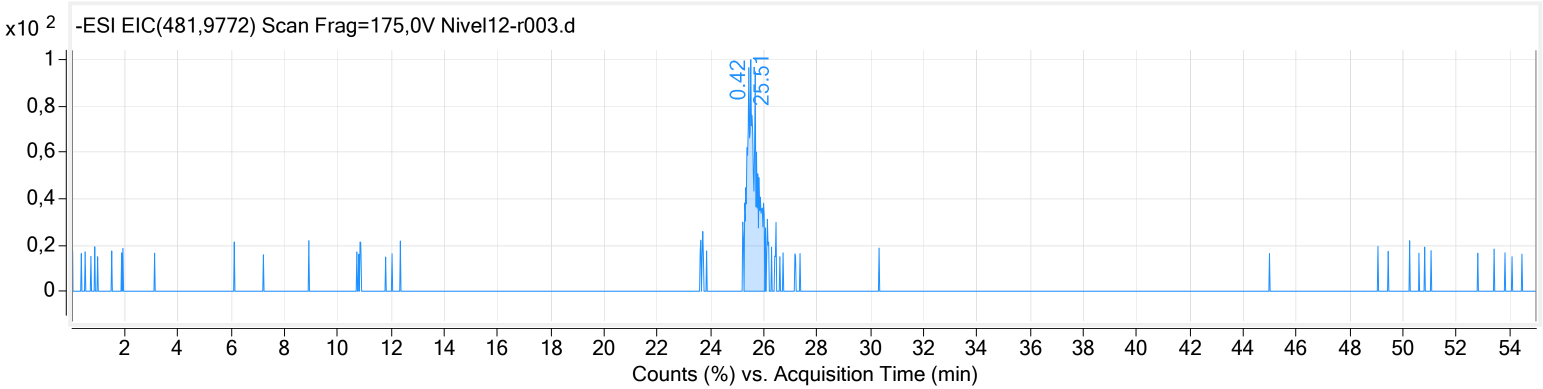

Supplementary Figure 4. ZIC-p-HILIC LC-MS chromatogram for some of *de novo* pyrimidine pathway analytes in tomato infected leave extract.

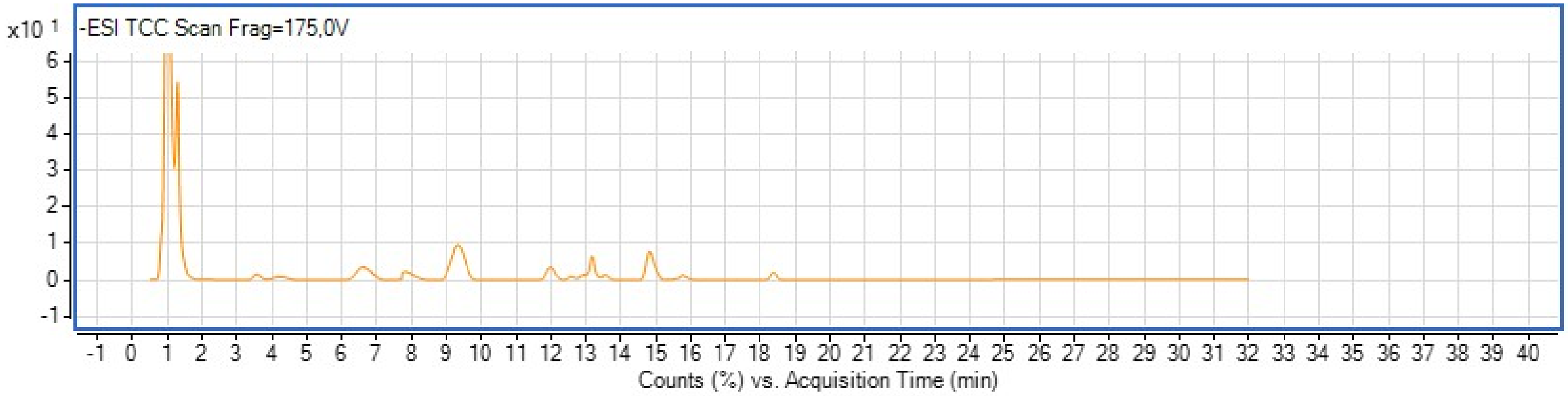

Supplement: Supplementary file 1 [file biomolecules-09-00328-s001.pdf]
